# Peripheral metabolic dysfunction is associated with cognitive frailty in a mouse model of type 2 diabetes

**DOI:** 10.1007/s11011-026-01956-z

**Published:** 2026-08-21

**Authors:** Maria Teresa Venuti, Federico Brandalise, Mattia Cappelletti, Attilio F. Speciani, Michela C. Speciani, Erica Cecilia Priori, Francesca Giammello, Elisa Roda, Paola Rossi, Daniela Ratto

**Affiliations:** 1 Department of Biology and Biotechnology “L. Spallanzani”, University of Pavia, Pavia, Italy; 2 Department of Biomedical Sciences, Division of Neuroscience and Clinical Pharmacology, University of Cagliari, Monserrato, Italy; 3 GEK srl, Milano, Italy; 4 Laboratory of Clinical & Experimental Toxicology, Pavia Poison Centre, National Toxicology Information Centre, Toxicology Unit, Istituti Clinici Scientifici Maugeri IRCCS, Pavia, Italy

**Keywords:** Type 2 diabetes, Methylglyoxal, Glycated albumin, Recognition memory, Hippocampus, High-fat diet, STZ-induced

## Abstract

Type 2 diabetes mellitus (T2DM) is a chronic metabolic disorder increasingly associated with cognitive impairment and neurodegeneration. Peripheral metabolic dysfunction, particularly insulin resistance and glycation stress, represents a critical link between diabetes and brain pathology. This study investigated the relationship between metabolic alterations and cognitive frailty using a murine T2DM model induced by a high-fat diet (HFD) combined with low-dose streptozotocin (STZ). Male C57BL/6J mice were longitudinally followed from adulthood to senescence and subjected to HFD alone or HFD plus STZ. Metabolic parameters, including fasting glycemia, oral glucose tolerance, insulin resistance (HOMA-IR), glycated albumin (GA), and methylglyoxal (MGO), were assessed over time. Cognitive function was evaluated using spontaneous behavioral tests of recognition memory (Novel Object Recognition, Object Location, and Y-maze), integrated into a cognitive frailty index. Post-mortem histological and immunohistochemical analyses were performed on pancreatic and hippocampal tissues. While HFD alone induced mild alterations, HFD/STZ mice developed a stable diabetic phenotype with severe hyperglycemia, pancreatic β-cell damage, elevated HOMA-IR, and increased GA and MGO levels. Notably, HFD/STZ mice exhibited a marked decline in recognition memory, affecting both knowledge and remembering components. At the central level, diabetic mice showed hippocampal cytoarchitectural alterations, increased tau hyperphosphorylation, and amyloid accumulation. Crucially, both insulin resistance and glycation stress (GA) directly correlated with recognition memory deficits. These findings demonstrate that peripheral metabolic dysfunction, i.e. insulin resistance and advanced glycation, directly correlates with hippocampal pathology and cognitive decline, supporting the translational relevance of the HFD/STZ model for diabetes-related cognitive frailty.

**Graphical abstract:**

**Figure:**
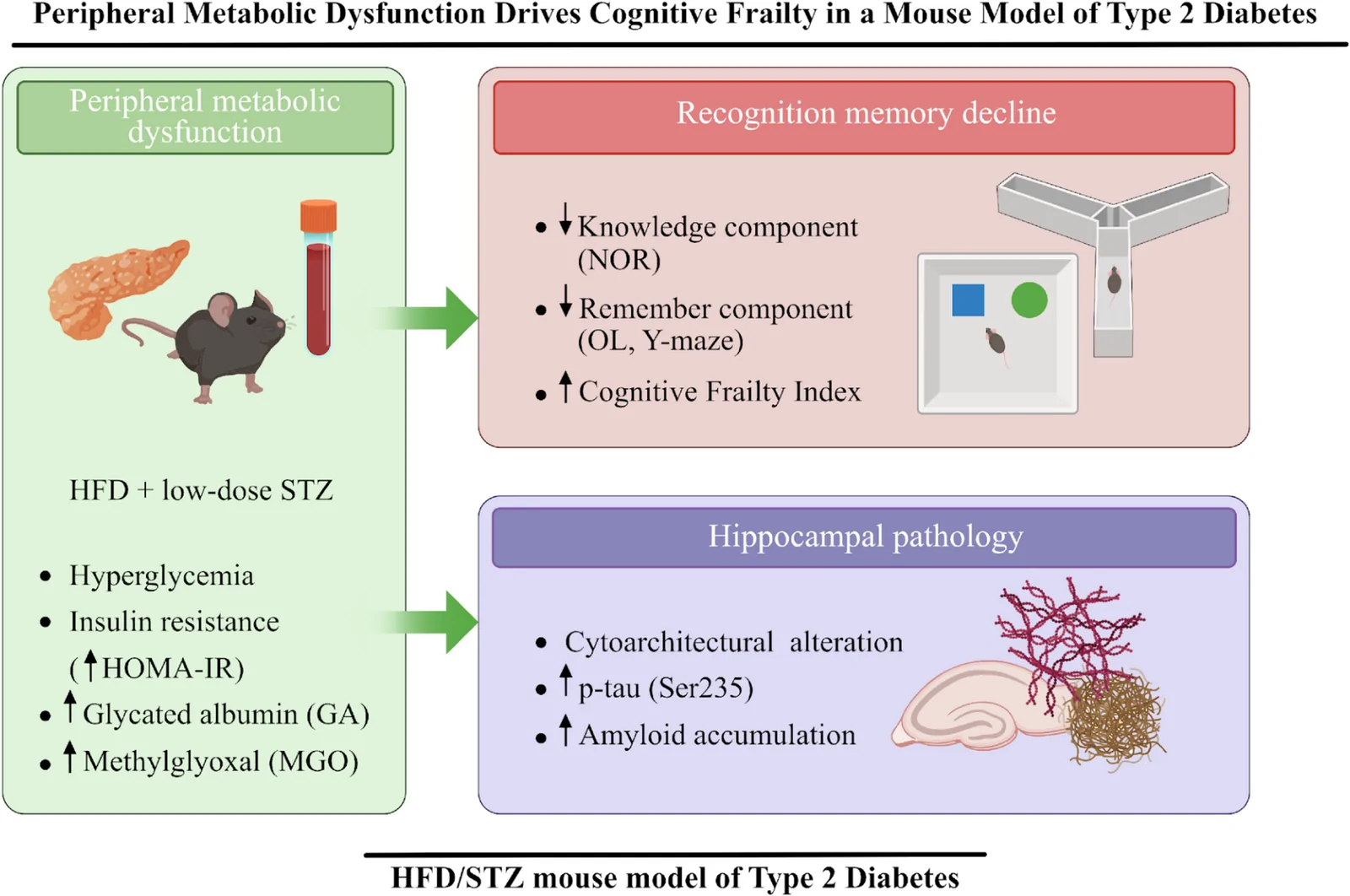

## Introduction

Type 2 diabetes mellitus (T2DM) is a chronic metabolic disorder, generally diagnosed in adulthood, characterized by insulin resistance, progressive β-cell dysfunction, and subsequent hyperglycemia. Accounting for about 90% of diabetes cases, T2DM is strongly associated with modifiable lifestyle factors such as a high-calorie diet, physical inactivity, and obesity. These factors not only contribute to the development of insulin resistance, also exacerbating systemic inflammation and dysregulating lipid metabolism, further impairing glucose homeostasis (Guthrie and Guthrie 2004; Nowotny et al. 2015).

Insulin resistance, a hallmark of T2DM, has emerged as a key pathophysiological mechanism linking peripheral metabolic dysfunction with cognitive impairment. Specifically, it compromises neuronal glucose uptake, alters synaptic plasticity, and promotes neuroinflammatory pathways, thereby potentially contributing to the early onset of neurodegenerative processes (Abdalla 2024; Gomez-Coral 2024).

Concurrently, the accumulation of glycation products has emerged as a crucial and additional metabolic alteration closely linked with diabetes-related complications. Biomarkers of glycation, such as glycated albumin (GA) and methylglyoxal (MGO), not only reflect glycemic control but are also implicated in dicarbonyl stress and advanced glycation end-product (AGE) formation, both of which may exacerbate metabolic and cognitive dysfunctions (Oliveira et al. 2024; Ayoub et al. 2025).

Together, these metabolic disturbances, specifically insulin resistance and glycation, set the stage for the onset of cognitive decline, which is increasingly recognized as a complication of T2DM. Epidemiological studies show that individuals with diabetes have a substantially higher risk of developing cognitive impairment and various forms of dementia compared to non-diabetic counterparts. Notably, the prevalence of mild cognitive impairment (MCI) among diabetic populations is estimated to range from 20 to 30%, with an elevated risk of progression to dementia (Wium-Andersen et al. 2020; Ortiz et al. 2022). Among the neurodegenerative conditions associated with diabetes, Alzheimer’s disease (AD) is one of the most extensively studied. Due to its strong metabolic component, some authors also refer to AD as “type 3 diabetes” (Michailidis et al. 2022; Kciuk et al. 2024).

In this perspective, prevention represents a critical strategy to mitigate the burden of type 2 diabetes mellitus and its associated cognitive complications. Early interventions aimed at preserving metabolic homeostasis, reducing insulin resistance, and limiting glycation-related stress may not only delay or prevent T2DM onset, but also attenuate downstream neurodegenerative processes, including those leading to Alzheimer’s disease. The identification of early metabolic biomarkers and experimental models that faithfully recapitulate the peripheral–central disease continuum is therefore essential for the development of effective translational preventive and therapeutic approaches.

Recognition memory, a type of long-term episodic memory, is commonly affected in diabetes-related cognitive decline. Behavioral tasks, such as novel object recognition (NOR), object location (OL), and Y-maze tests, are widely used in rodents to evaluate recognition and spatial memory (Patel et al. 2016; Farajpour et al. 2017; Vilela et al. 2023; Gao et al. 2023).

Streptozotocin (STZ), an antibiotic that selectively destroys pancreatic β-cells, is widely used to generate experimental Diabetes Mellitus (DM) models. Literature describes various protocols differing in STZ dosage and its combination with other molecules or specific diets. In particular, a high-dose STZ regimen induces complete destruction of pancreatic β-cells, reproducing T1DM. On the contrary, a low-dose STZ protocol induces a gradual disruption of pancreatic β-cells, mimicking the pathogenesis of T2DM. Among T2DM models, the combination of HFD and low-dose STZ is particularly valuable, as it reproduces both insulin resistance and partial β-cell dysfunction, thereby closely resembling the progressive nature of human T2DM. Compared to genetic models, this approach better mirrors the interplay between dietary factors, metabolic impairment, and cognitive decline (Singh et al. 2024; Brito et al. 2025).

In the present study, a T2DM animal model was established by administering a HFD combined with intraperitoneal STZ injection (HFD/STZ) to investigate the complex mechanisms underlying the relationship between T2DM and cognitive decline. This work focuses on several key points, including the association between peripheral metabolic parameters, insulin resistance, and cognitive performance.

## Materials and methods

### Animals

Fourteen ten-month-old wild-type male mice (strain C57BL/6J) were maintained in single cages in the Animal Care Facility at the University of Pavia under a 12-hour light/dark cycle. Water and food were provided *ad libitum*. All experimental procedures were carried out following institutional welfare committee guidelines and were approved by the Ethics Committee of the University of Pavia (Ministry of Health, License number 220/2022-PR), in compliance with the European Council Directive 2010/63/EU on the protection of animals used for scientific purpose.

Mice were initially fed a standard diet (normal diet, ND), consisting of 4RF21 pellets supplied by Mucedola Srl. The ND comprised 42.63% kcal carbohydrates, 18.50% kcal protein, and 3% kcal fats. Following a two-weeks acclimatation period on the ND, all animals were shifted to a high-fat diet (HFD), in which 59% of total caloric intake was derived from fats. HFD pellets were obtained from the Laboratory of Dr. Piccioni Srl (Gessate, MI, Italy) and consisted of 20% kcal from carbohydrates, 15% from kcal protein, and 59% kcal from fat.

### In vivo study

**Fig. 1:**
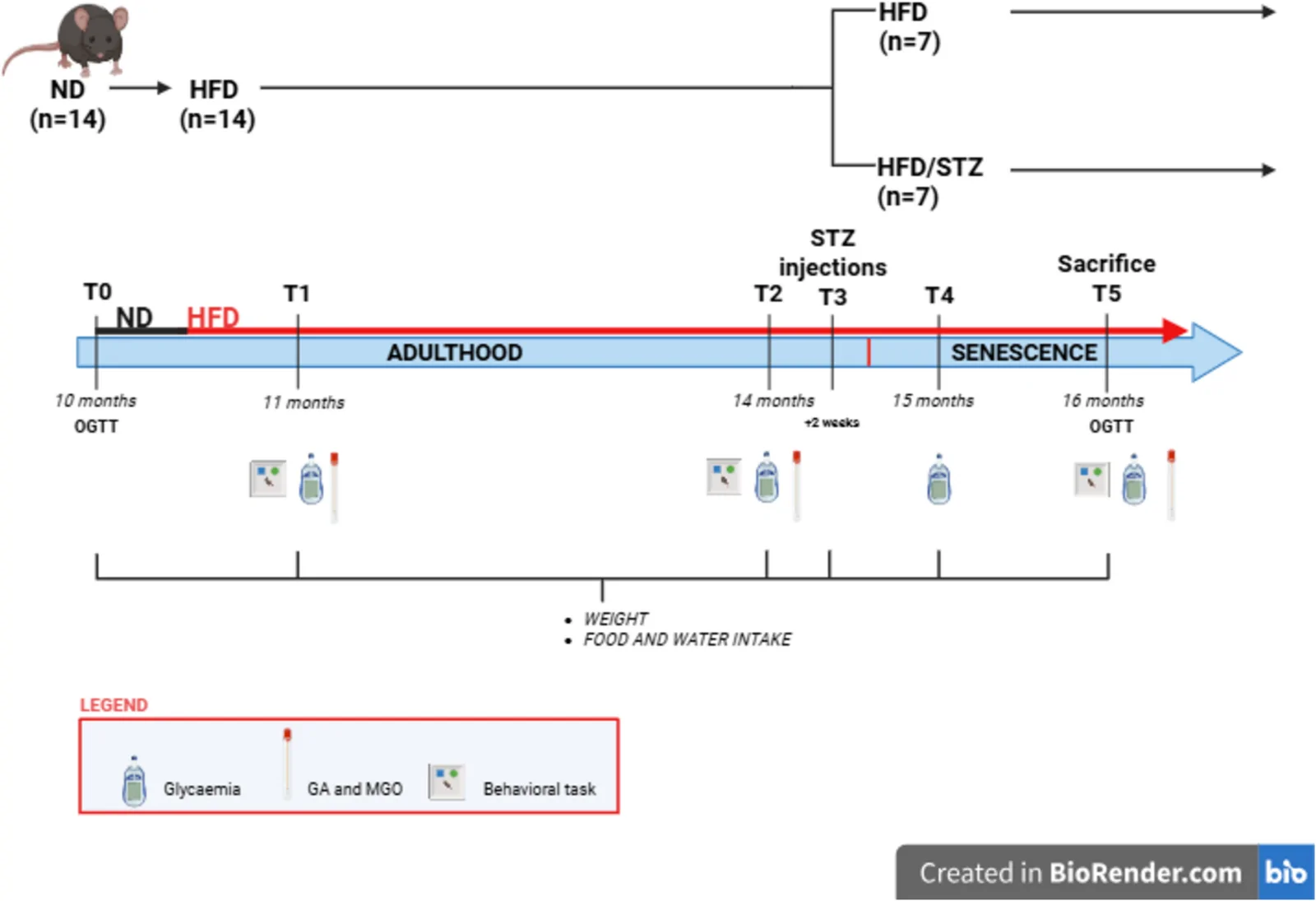
Experimental plan

The experimental design consisted of five time points (Fig. 1), starting from 10 months (T0) and extending to 16 months (T5) of mouse age. At T0, all animals previously fed with the ND were shifted to HFD. At this time, Oral Glucose Tolerance Test (OGTT) and fasting glycemia were performed. At T1, corresponding to one month after HFD initiation, fasting glycemia, glycated albumin (GA), and methylglyoxal (MGO) were measured, and spontaneous behavioral tests were performed. At T2, two weeks prior to the streptozotocin (STZ) injection, fasting glycemia, GA, and MGO were assessed, and spontaneous behavioral tests were conducted. At T3 (14 months of age), animals were randomly assigned to two experimental groups: (i) 7 control animals fed with HFD received intraperitoneal (i.p.) injections of physiological saline (0.9% NaCl) for five consecutive days (HFD group), while (ii) 7 mice received STZ i.p. injections to induce diabetes (HFD/STZ group; see detailed induction protocol in the following paragraph). One-month post-injections (T4), fasting glycemia was assessed in all mice to confirm successful diabetes induction. At T5 (16 months of age), fasting glycemia, GA and MGO were monitored, and the OGTT and spontaneous behavioral tests were performed. All animals were sacrificed at T5; blood and organs were collected as described (see details in the following paragraphs). Body weight, as well as food and water consumption, were monitored at all experimental times throughout the entire experimental period.

### Murine T2DM induction and measurement of fasting glycemia, GA, and MGO

Based on a previous study, the T2DM model was induced using HFD/STZ combination (Furman 2021). After being fed with HFD for four months (T3), the HFD/STZ group of mice received an intraperitoneal injection of 50 mg/kg STZ for five consecutive days (dissolved in 100 mM citrate buffer solution). The HFD group received physiological saline (0.9% NaCl). Specifically, the HFD/STZ mouse was defined as diabetic if the fasting blood glucose exceeded 250 mg/dL (Furman 2021; Peng et al. 2021; Chen et al. 2024), a threshold met by all mice in the study. For longitudinal metabolic assessments (T0, T1, T2, and T5), animals were fasted at least 4 h prior to blood collection, in order to obtain a stable metabolic baseline for the measurement of glycemia, glycated albumin (GA), and methylglyoxal (MGO). Blood was collected from the tail vein, and a single drop was used for glucose measurements. Glucose levels were determined using a reactive test strip with a OneTouch Verio Reflect^®^ glucometer (Life scan Italy Srl). To evaluate GA and MGO levels, 200 µL of blood was collected using synthetic swabs (Copan 552 C, purchased from Diagnostic International Distribution S.p.A.) and analyzed by the GEK Lab laboratory using a specific ELISA kit (Venuti et al. 2025).

### Measurement of OGTT

OGTTs were conducted at time points T0 and T5. The mice were fasted overnight and subsequently administered a glucose solution via oral gavage (Glucosio Sclavo, diagnostics 75 g/150 ml) at a dosage of 1 g/kg body weight (Ayala et al. 2010; Nagy and Einwallner 2018). Blood glucose levels were measured at 0 min (fasting glycemia) and 30-minute intervals post-glucose administration, until 3 h. Glucose response curves and the corresponding area under the curve (AUC) were calculated for each mouse tested using ORIGIN 6.0 software. Mean values and SEM for both glucose curves and AUCs were subsequently derived.

### Insulin determination

At T5, all mice were fasted for at least 4 h, and blood samples were collected. After centrifugation at 1500 RCF for 10 min, the serum of the blood samples was separated and insulin levels were then measured using the Mouse/Rat INS (insulin) ELISA kit (EMR0002, FineTest, Labclinics, Barcelona, Spain). Insulin resistance was assessed using the homeostasis model of insulin resistance (HOMA_IR) index, calculated with the following formula:

$$\:\begin{array}{c}HOMA\_IR=\\\:\frac{fasting\:plasma\:glucose\:\left(\frac{mmol}{L}\right)\times\:fasting\:plasma\:insulin\:\left(\frac{\mu\:IU}{mL}\right)}{22.5}\end{array}$$

### Behavioral test and cognitive frailty index

Spontaneous behavioral tests were performed to evaluate recognition memory in mice. At selected timepoints, i.e., T1, T2, and T5, all animals performed the Novel Object Recognition (NOR) test, a well-established method for studying the “Knowledge component” of recognition memory. This task refers to the ability to identify a new object in the environment and is comparable to the “Stenberg Item Recognition paradigm” used in clinical practice. Additionally, at the same timepoints, the Object Location (OL) and Y-maze tasks were performed in all mice to investigate the “Remember component” of recognition memory, which involves recalling the spatial and temporal context of a stimulus, similar to the “Four mountains test and “Image-location memory task” employed in clinical diagnostics. Mouse behavior was recorded using a SMART video tracking system (2 Biological Instruments, Besozzo, Varese, Italy) with a selected sampling time of 40 ms/point, and monitored by a Sony CCD color video camera (PAL). To avoid potential learning effects in the behavioral tests, long intervals between sessions, as well as different objects and/or spatial configurations, were used at each testing session (Ennaceur and Delacouret 1988; Antunes et al. 2012; Leger et al. 2013).

#### Novel object recognition (NOR), object location (OL), and Y-maze tests

The NOR, OL, and Y-maze behavioral tests were carried out as previously described (Brandalise et al. 2017; Rossi et al. 2018; Ratto et al. 2019; Roda et al. 2022). Briefly, the NOR and OL tasks consisted of three sequential phases: habituation, familiarization, and test. During the test phase, both the number and the duration of the approaches with the novel and familiar objects or locations were measured as cognitive parameters. To evaluate the discrimination between novel and familiar objects, the Mean Novelty Discrimination Index (DI) was calculated according to the following formula (Silvers et al. 2007),

$$\:DI=\left(n-f\right)/\left(n+f\right)$$

where (n) represents the average time or number of approaches to the novel or relocated object and (f) corresponds to the average time or number of approaches to the familiar one. The DI values ranged from − 1 to 1, where − 1 means a complete preference for the familiar object, 0 means no preference, and 1 means a complete preference for the novel or relocated object.

In particular, total object exploration time and number were monitored longitudinally during each session to verify that all animals explored the objects sufficiently for reliable discrimination index calculation. Notably, no significant differences in total exploration time or number were observed between groups, indicating that diabetic mice explored the objects sufficiently and that the observed differences in discrimination performance are unlikely to be explained by reduced exploration activity.

In the Y-maze test, each mouse was placed at the center of the apparatus and allowed to explore all three arms freely for 8 min. Spontaneous alternation behavior was evaluated by observing the pattern in which the mouse fully entered each arm (with its hind paws completely crossing into the arm). Alternation of triplet was defined as the number of consecutive entries into all three arms on overlapping triplet sets, and the alternation (%) was calculated following this formula:

$$\:\begin{array}{c}Alternation\:\left(\%\right)=\\\:\left[\right(actual\:alternation)/(Total\:number\:of\:arms\:entries-2\left)\right]\times\:100\end{array}$$

#### Global frailty index

To obtain an integrated measure of recognition memory performance, a cognitive frailty index (FI) was calculated by combining the results of the behavioral tests. The FI was designed to reflect the two complementary components of recognition memory: the “knowledge component” (evaluated using NOR test), and the “remember component” (evaluated using OL and Y-maze tests). For each parameter of each behavioral test, an individual frailty score was first calculated based on the relative decline in performance compared to the reference condition, according to the following formula (Parks et al. 2012; Ratto et al. 2019):

$$\:FI=(observation\:value-Mean\:Value\:at\:T0)/\left(SD\:at\:T0\right)*0.25$$

Subsequently, for each behavioral test, a single frailty index was obtained by averaging the frailty scores of all parameters derived from that test. Next, to avoid overrepresentation of any single cognitive domain, the “remember” component was obtained by averaging the frailty scores derived from the OL and Y-maze tests, and then, the global cognitive frailty index was calculated by averaging the “knowledge” and “remember” components. With this approach, the two main domains of recognition memory contribute equally to the final FI, despite being assessed by a different number of behavioral tests. Higher FI values indicate a greater decline in cognitive performance. This integrated parameter allows a more comprehensive evaluation of cognitive frailty by reducing variability associated with individual tests and capturing the multidimensional nature of recognition memory.

### Ex vivo tissue sampling preparation

At T5, all mice were anesthetized with isoflurane inhalation (Aldrich, Milwaukee, WI, USA) prior to decapitation. Pancreas and brains were quickly excised and collected, rinsed in 0.9% NaCl saline solution, and fixed in 4% paraformaldehyde dissolved in 0.1 M phosphate buffer (pH 7.4). Specifically, pancreases were fixed for 7 h, while brains were fixed for 48 h, at room temperature. All tissues underwent post-fixation for an additional 1.5 h at 4 °C in the same fixative solution. Subsequently, a dehydration step was performed by immersion in absolute ethanol for 1 h, followed by acetone. Finally, samples were embedded in Paraplast X-TRA (Sigma Aldrich, Milan, Italy). Coronal serial sections were cut using a manual rotary microtome, at a thickness of 6 μm and 8 μm for pancreas and brains, respectively. Sections were then collected on silane-coated slides. Ex vivo tissue analysis was conducted on 3 mice per group, randomly selected from each experimental cohort. A minimum of 4 pancreatic and 4 brain sections were evaluated per mouse.

#### Light microscopy: Hematoxylin and Eosin staining (H&E)

H&E staining was performed to examine the histopathological features and identify potential structural alterations in the pancreas and hippocampus, following established protocols (Roda et al., 2022; De Luca et al., 2023).

Given the complex architecture and functional specialization of both pancreas and central nervous system (CNS), H&E staining provided a clear overview of tissue organization and delineated region-specific histological characteristics (Roda et al., 2023; Longnecker, 2021). Brightfield microscopy at low magnification enables clear identification of distinct areas within the pancreas and the hippocampus. Whereas coronal plane and subregional anatomy of the hippocampus were readily visualized, the coronal orientation of the pancreas was only confirmed based on the presence of rounded pancreatic ducts lined with cuboidal epithelium and the distribution of the islets of Langerhans. Histological sections were examined using a Leica DM6B widefield microscope (Leica Microsystems, Buccinasco, MI, Italy); digital images were captured with a Leica DFC 7000 T CCD camera (Leica Microsystems, Buccinasco, MI, Italy) and analyzed using the Leica Application Suite X (LAS X) software (Version 5.1.0). The whole hippocampus was reconstructed using the LAS X Navigator imaging system, including the merge function.

##### **Insulitis scoring in pancreatic sections**

To assess possible lymphocytic infiltration within the Langerhans islets parenchyma, pancreatic tissue sections were analyzed. To determine the presence and severity of insulitis, a minimum of 30 islets per mouse (evaluated across at least 4 tissue sections per animal, yielding 90 (30 × 3 mice) islets total per experimental group) were evaluated. All evaluations were conducted under double-blinded conditions. The grading criteria for insulitis were established as follows: normal islets received a score of 0; perivascular or periductal infiltration was scored as 1; peri-insulitis was graded as 2; mild insulitis, where less than 25% of the islet was infiltrated, was assigned a score of 3; severe insulitis, with more than 25% of the islet infiltrated, received a score of 4 (Pejnovic et al., 2013; Pavlovic et al. 2018). The values were averaged to give final scores. Next, insulitis was quantified as a weighted score by multiplying the number of islets in each infiltration category by the corresponding score (0–4), summing these values, and normalizing by the total number of islets analyzed for each animal. The weighted score was used for correlating insulitis with insulin resistance (i.e., HOMA-IR).

##### **Hippocampal Injury Evaluation**

For histopathological evaluation, as previously reported, four representative sections per mouse (n = 3 per group) were examined, focusing on the Dentate Gyrus (DG) and Cornu Ammonis (CA) regions of the hippocampus. Quantitative assessments included: (i) the total thickness of the DG granule cell layer, (ii) the thickness of the pyramidal cell layer within the CA subregions, and (iii) cell density (cells/mm²) calculated within a 300 × 300 μm region of interest (ROI) for each area. For each hippocampal section, the mean value of each parameter was determined and the measurements for each section were ultimately averaged to obtain the mean value per animal.

#### Immunohistochemistry

The immunohistochemistry was performed on 4 sections per mouse (*n* = 3 per group), as previously reported. After deparaffination in xylene (Carlo Erba, Cornaredo, Italy), sections were rehydrated in a series of decreasing ethanol concentrations and rinsed in phosphate-buffered saline (PBS, Sigma-Aldrich, Milan, Italy). The hippocampal slides were incubated at RT for 7 min in a blocking buffer for the suppression of endogenous peroxidases (3% H2O2 in 10%methanol in PBS), then for 20 min in fetal calf serum to block non-specific antigen binding sites. Immunohistochemistry was performed using commercial antibodies on mouse hippocampal sections to localize the presence and distribution of two specific markers involved in Alzheimer’s pathology: (i) Anti-Beta (β)-Amyloid (AβPP), and (ii) Anti-phospho-Tau (Ser235) (details and dilutions are reported in Table 1). The sections were incubated at 4 °C overnight in a dark moist chamber. Subsequently, slides were incubated with biotinylated secondary antibodies (Vector Laboratories, Burlingame, CA, USA) for 30 min and horseradish peroxidase conjugated avidin-biotin complex (Vector Laboratories, Burlingame, CA, USA) for 30 min at RT. Then, 0.05% 3,3-diaminobenzidine tetrahydrochloride (DAB; Sigma Aldrich, Milan, Italy) with 0.01% H2O2 in Tris–HCl buffer (0.05 M, pH 8) was used as a chromogen, followed by nuclear counterstaining with Haematoxylin. Then, sections were dehydrated in ethanol, cleared in xylene (Carlo Erba Reagents, Cornaredo, Italy), and finally mounted in Eukitt (Kindler, Freiburg, Germany). For control staining, primary antibody was omitted in some sections, which were incubated with phosphate-buffered saline only. No immunoreactivity was observed under these conditions.

**Table 1:** Primary and secondary antibodies used for immunohistochemical reactions

	Antigen	Manufacturer, Species, Mono-Polyclonal, Catalogue or Lot No., RRID	Dilution
Primary antibodies	Anti-Beta (β)-Amyloid	Sigma-Aldrich (St. Louis, MO, USA), Mouse monoclonal IgG1, clone 22C11, Cat# MAB348; RRID: AB_94882	1:100
	Anti-phospho-Tau (Ser235)	Sigma-Aldrich (St. Louis, MO, USA), Mouse monoclonal IgG1k, clone RN235, Cat# MABN2275; RRID: AB_3738554	1:500
Secondary antibodies	Biotinylated horse anti-mouse IgG	Vector Laboratories (Burlingame, CA, USA), Horse, Cat# PK-6102, RRID: AB_2336821	1:200

##### **Quantitative analysis**

For the quantitative assessment of selected markers expression, the immunopositivity was analyzed across four hippocampal subregions per Sect. (4 sections per mouse; 3 mice per experimental group): the molecular layer of CA1 (ML-CA1), the pyramidal layer of CA1 (P-CA1), the pyramidal layer of CA3 (P-CA3), and the molecular layer of the dentate gyrus (ML-DG).

Sections were observed by Leica DM6B WF microscope (Leica Microsystems, Buccinasco, MI, Italy). Images were acquired with a Leica dfc 7000 t CCD camera (Leica microsystems, Buccinasco, MI, Italy) and stored on a PC running the Leica Application Suite X (LAS X) software (Version 5.1.0). Furthermore, three sections per animal were analyzed using ImageJ (ImageJ 1.46p, NIH, Bethesda, MA, USA), and for each animal the mean and standard error of the mean (SEM) were calculated.

For p-Tau evaluation, bright-field images were inverted to render immunopositive regions lighter than the background or non-stained neurons, and background intensity and hematoxylin counterstaining were subtracted. Pixel density in immunopositive regions was then quantified and expressed as optical density (OD). For each section, OD was measured in 10 rectangular regions of interest (ROIs, 40 × 40 μm) in the molecular layers and in 10 circular ROIs (30 × 30 μm) in the pyramidal layers, with background OD subtracted from each measurement; ROI dimensions and image magnification were kept constant across all analyses. The mean value of each section was averaged to obtain the mean value per animal.

For amyloid precursor protein (APP) evaluation, identified through the β-amyloid–reactive antibody, plaque area and the number of APP-positive spots were quantified using the “polygon selection” and “multi-point” tools, respectively, and APP plaque density was calculated according to the formula:

  

$$\:number\:of\:single\:APP\:spots/area\:plaque$$

### Statistical analysis

Data are presented as mean ± standard error of the mean (SEM). The statistical analysis of Kaplan-Meier graphs was performed using a Log-rank (Mantel-Cox) test. To evaluate the statistical differences among the time and experimental groups one-way ANOVA, two-way ANOVA (followed by the Bonferroni post-hoc test) or unpaired student t-test were used according to the distribution of the data and the number of experimental groups. In addition, Spearman’s rank correlation analysis was performed to assess associations between metabolic, behavioral, and biochemical parameters. Microsoft Excel and Prism 5 (GraphPad Software, San Diego, CA, USA) were used for conducting statistical analysis. Statistical significance was assigned as follows: *P* < 0.05 (*), *P* < 0.01 (**), *P* < 0.001 (***).

## Results

The study longitudinally monitored mice starting from adulthood (10 months, T0) to senescence phase (16 months, T5). At T0, while maintained on a standard diet of the animal care facility (referred as normal diet, ND), fasting glycemia was measured, and oral glucose tolerance test (OGTT) was performed in all mice. After these assessments, all animals were switched to a high-fat diet (HFD). Subsequently, after 1 month, at T1 (11 months), fasting glycemia, GA, MGO, and behavioral tests were conducted and were repeated at T2 (14 months). At T3, mice were randomly divided into two groups: one received a physiological saline injection (HFD), while the other received STZ injections to induce diabetes (HFD/STZ), as detailed in Materials and Methods. Fasting glycemia was measured at T4 (15 months) to verify the effect of STZ injection. At T5 (16 months), a final assessment was conducted, including fasting glycemia, GA, MGO, OGTT assessment, and behavioral tests. Finally, at T5, mice were sacrificed, and blood and organs were collected. Body weight, food, and water intake were monitored throughout the experiment (for more details, see the Materials and Methods section).

### HFD alone and HFD/STZ combination effects on survival, body weight, food and water intake, fasting glycemia, glycated albumin and methylglyoxal levels

**Fig. 2:**
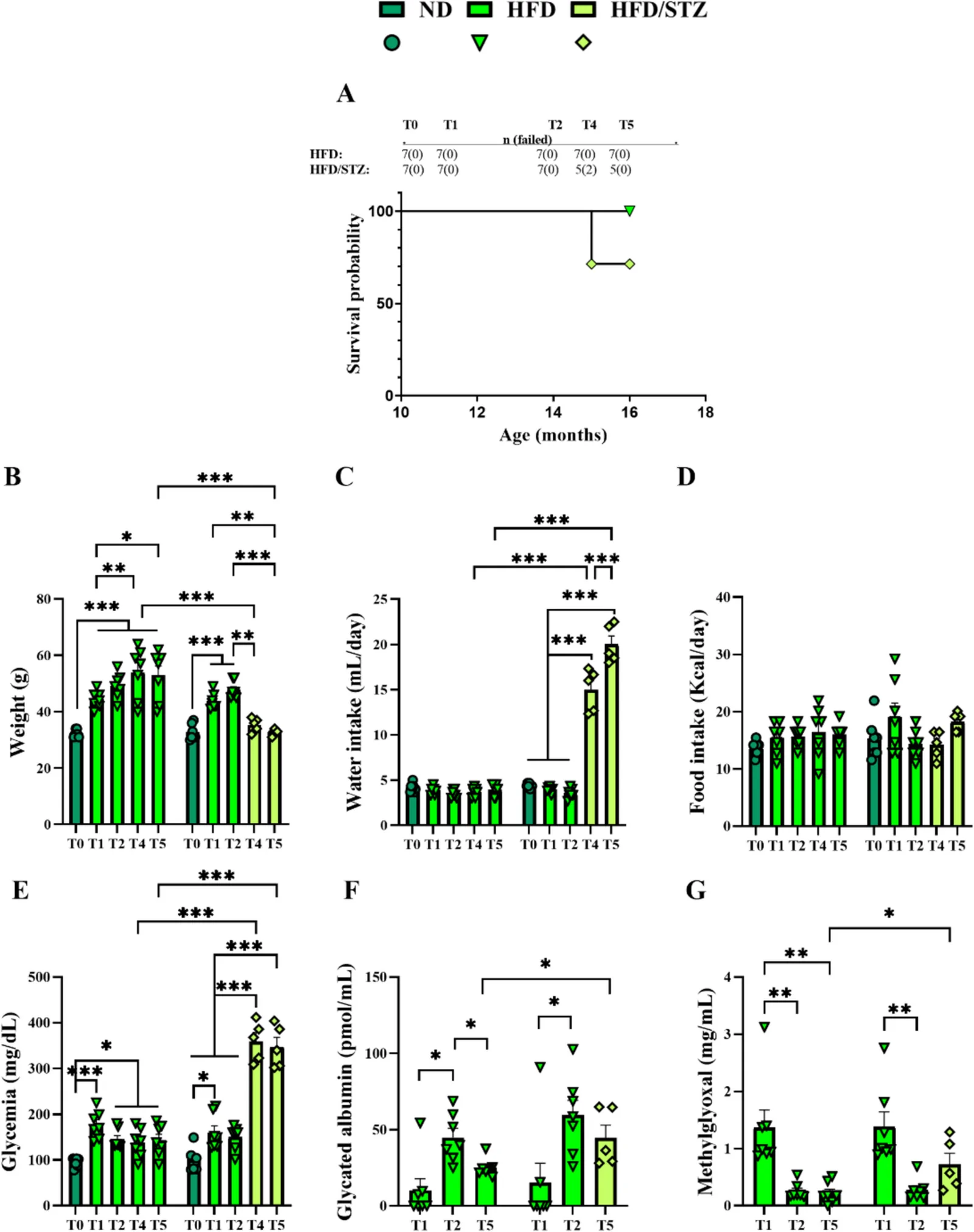
Time-dependent effects of HFD and HFD + STZ on survival, body weight, food and water intake, and metabolic parameters. Survival probability, body weight, water and food intake, glycemia, GA and MGO levels were evaluated in HFD (green triangle) and HFD/STZ (light green rhombuses) mice at different timepoints. In panels B, C, D, E, F and G, the ND group (dark green circle) at T0 represents baseline measurements obtained from the same cohort of mice before allocation to the HFD and HFD + STZ groups and before the respective treatments. (**A**) Kaplan–Meier survival curves, (**B**) Body weight (g), (**C**) Water intake (ml/day), (**D**) Food intake (Kcal/day), (**E**) Fasting glycemia (mg/dl); (**F**) glycated albumin (GA, pmol/ml) and (**G**) methylglyoxal (MGO, mg/ml). Data are expressed as mean ± SEM. Statistical significance for Kaplan-Meier analysis was obtained with a Log (Mantel-Cox) rank test. Statistical significance for all other parameters was obtained using Two-Way ANOVA followed by Bonferroni post-hoc test): *p* < 0.05 (*); *p* < 0.01 (**); *p* < 0.001 (***)

All mice were longitudinally monitored from adulthood (T1) to senescence (T5), with survival and metabolic parameters assessed throughout the study. Survival probability was evaluated by Kaplan–Meier analysis across all experimental conditions during the animals’ lifespan (Fig. 2A). No statistically significant differences were observed between the HFD and HFD/STZ groups, indicating that STZ administration did not affect survival up to 16 months of age.

In addition, body weight (Fig. 2B), food intake (Fig. 2C), and water consumption (Fig. 2D) were monitored over time in both experimental groups. All data were compared to T0, which represents the experimental time when animals were still fed the normal diet (ND) before starting the HFD (see Fig. 1).

In the HFD group, the body weight showed a gradual and statistically significant increase at T1 (44.29 ± 1.13 g, *n* = 7, *p-value* < 0.001), T2 (49.14 ± 1.13 g, *n* = 7, *p-value* < 0.001), T4 (53.86 ± 3.45 g, *n* = 7, *p-value < 0.001*) and T5 (52.86 ± 3.32 g, *n* = 7, *p-value < 0.001*) compared to that measured in the ND group at T0 (32.29 ± 0.47 g, *n* = 7). The body weight gain assessed from T1 to T4 was statistically significant (T1 vs. T4, *p-value* < 0.01). At T4 and T5, the body weight reached a steady state value. Regarding water and food intake, the HFD group showed stable water consumption across all time points analyzed (about 3–5 ml/day; Fig. 2C), and food intake significantly decreased over time in the HFD group when consumption was expressed as g/day (data not shown), but no changes were observed when it was expressed as kcal/day (Fig. 2D), remaining within a mean range of about 14 to 16 kcal/day.

After characterizing the longitudinal effect of mice fed with HFD, the additional impact of STZ-injections (HFD/STZ) on body weight, water and food intake was assessed. As expected, in the HFD/STZ mice, the body weight significantly decreased at T4 (35.4067 ± 1.080.92 g, *n* = 5) and T5 (32.80 ± 0.49 g, *n* = 5) compared to HFD mice at the same experimental time points (*p-value* < 0.001, Fig. 2B). Furthermore, in HFD/STZ mice, a marked and statistical increase in water intake was observed at T4 (15.00 ± 1.04 ml/day, *n* = 5), and T5 (20.00 ± 0.94 ml/day, *n* = 5) compared to T4 and T5 in HFD mice (*p-value* < 0.001). Notably, water intake in HFD/STZ mice significantly increased by over 29% from T4 to T5 (*p-value* < 0.001, Fig. 2C). No significant differences in food intake were observed between HFD/STZ and HFD mice at T4 and T5, suggesting that STZ induction did not affect food intake (Fig. 2D).

These findings indicate that STZ administration in HFD-fed mice induced body weight loss and polydipsia, suggesting the development of a diabetic metabolic phenotype.

To further characterize the metabolic phenotype of the experimental groups, fasting glycemia, glycated albumin (GA), and methylglyoxal (MGO) levels were subsequently assessed.

At T0, the fasting glucose level in ND mice was euglycemic (about 100 mg/dl). In HFD mice, fasting glucose was measured at T1 (176.86 ± 11.30 mg/dl, *n* = 7), T2 (145.43 ± 8.08 mg/dl, *n* = 7), T4 (138.00 ± 11.90 mg/dl, *n* = 7), and T5 (143.29 ± 12.97 mg/dl, *n* = 7). All values were statistically higher compared to T0 (*p-value* < 0.001 vs. T1 and *p-value* < 0.05 vs. T2, T4, and T5). No statistically significant differences were measured among the different time points in the HFD group (Fig. 2D). These results indicate that, after 1 month, HFD alone induces a rapid and sustained increase in fasting glycemia, which remains stable through six months. In the HFD/STZ mice, fasting blood glucose levels were significantly increased both one (T4, 359.60 ± 19.25 mg/dl, *n* = 5) and two months (T5, 347.60 ± 20.64 mg/dl, *n* = 5) after STZ injections, compared to those assessed in HFD mice at the same experimental timepoints (*p-value* < 0.001, Fig. 2E). According to the glycemic value threshold reported in the literature (250 mg/dl, Furman 2021; Chen et al. 2024), after STZ induction all mice were found to be diabetic mice.

The mean GA (Fig. 2F) value statistically increased in HFD mice at T2 (44.84 ± 5.98 pmol/mL, *n* = 7) compared to T1 (10.301 ± 7.51 pmol/mL, *n* = 7, *p-value* < 0.05), but decreased at T5 (24.92 ± 2.23 pmol/mL, *n* = 7, *p-value* < 0.05 vs. T2, *p-value =* NS vs. T1), suggesting a time-dependent metabolic adaptation to HFD feeding. The mean MGO (Fig. 2G) value statistically decreased in HFD mice at T2 (0.25 ± 0.05 µg/mL, *n* = 7) and T5 (0.22 ± 0.07 µg/mL, *n* = 7), compared to T1 (1.37 ± 0.30 µg/mL, *n* = 7, *p-value* < 0.001).

Interestingly, in HFD/STZ mice, the mean GA value at T5 (44.65 ± 8.35 pmol/mL, *n* = 5) was significantly higher than that observed in HFD mice at the same age (*p-value* < 0.05), and was comparable to that measured at T2 (Fig. 2F). Furthermore, the HFD/STZ mice showed a significantly increased MGO mean value at T5 (0.72 ± 0.20 µg/mL, *n* = 5) compared to that determined in HFD mice at the same experimental point (*p-value* < 0.05, Fig. 2G).

Overall, these findings indicate that STZ administration in HFD-fed mice successfully induced a diabetic phenotype, characterized by sustained hyperglycemia, increased GA and MGO levels, and the development of body weight loss and persistent polydipsia, both recognized consequences of diabetic hyperglycemia.

### Pancreatic Islet inflammation induced by HFD and STZ

To estimate the potential alterations induced by HFD diet alone or in combination with STZ (HFD/STZ) on pancreatic integrity, semi-quantitative histopathological examinations were performed on pancreatic sections from HFD and HFD/STZ mice at T5, after H&E staining.

A scoring system was utilized to evaluate the extent of tissue damage using conventional brightfield microscopy according to a semiquantitative scale ranging from undetectable/normal (-) to severe/striking (4) (Pavlovic et al. 2018). Specifically, 30 pancreatic islets for each experimental group were analyzed.

The degree of lesions was recorded and graded as follows: score 0, normal islet architecture; score 1, perivascular/periductal infiltration; score 2, peri-insulitis; score 3, mild insulitis (< 25% of the islet infiltrated); score 4, severe insulitis (more than 25% of the islet infiltrated).

**Fig. 3:**
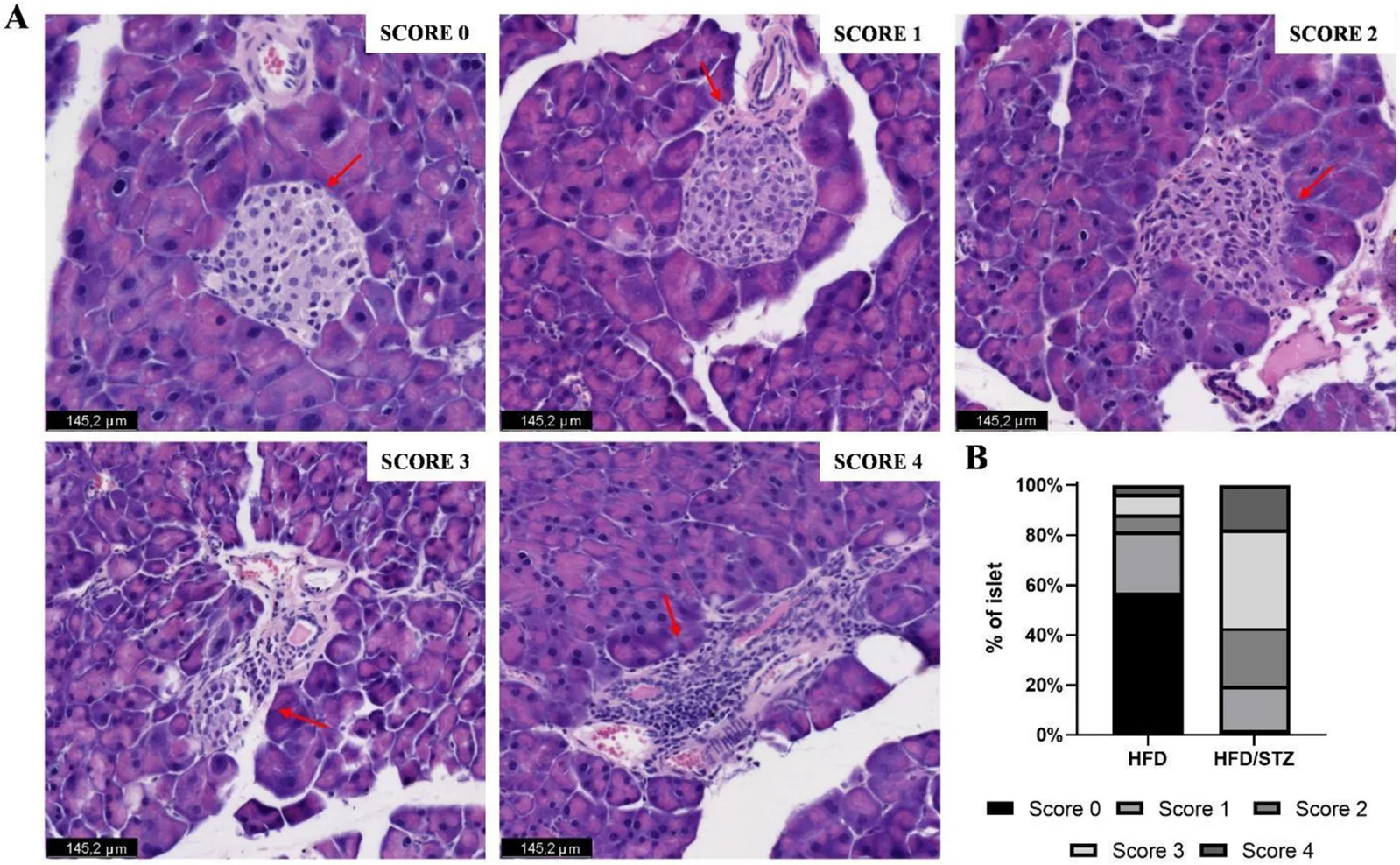
Pancreatic Islet Insulitis in HFD and HFD/STZ mice at T5. Histological characterization by H&E staining. (**A**) Representative micrographs showing pancreas histological architecture from different groups. Red arrows highlight characteristic features associated with each score. Light microscopy magnification: 40x. Scale bars 145.2 μm. (**B**) Semi-quantitative scale ranging from undetectable (0) to severe (4) tissue damage. In particular, the injury degree was recorded and graded as follows: 0, normal islet; 1, perivascular/periductal infiltration; 2, peri-insulitis; 3, mild insulitis (< 25% of the islet infiltrated); 4, severe insulitis (more than 25% of the islet infiltrated)

The analysis revealed largely preserved pancreatic islet morphology in the HFD group, with 56.98% of the islets classified as normal (score 0). In contrast, the HFD/STZ group exhibited a drastic reduction in normal islets, with only 1.78% classified as score 0, indicating significant structural damage (Fig. 3A and B).

The percentage of perivascular/periductal infiltration (score 1) was more frequently observed in the HFD group (24.42%) compared to the HFD/STZ group (17.86%). Conversely, peri-insulitis (score 2) was more prevalent in the HFD/STZ group (23.21%) compared to the HFD group (6.98%). Similarly, mild insulitis percentage (score 3) was predominantly detected in the HFD/STZ group (39.28%) compared to HFD (8.14%). Finally, the percentage of severe insulitis (score 4) was higher in HFD/STZ animals (17.86%) compared to HFD mice (3.49%, Fig. 3A and B).

These results confirm that the combination of HFD and STZ induces more pronounced and extensive pancreatic damage compared to the effects of HFD alone, as reflected by the higher occurrence of advanced insulitis scores in the HFD/STZ group.

### Effects of HFD and HFD/STZ on recognition memory

To investigate the potential link between changes in metabolic parameters, diabetes development, and recognition memory, specific behavioral tests at selected experimental timepoints to assess long-term episodic recognition memory (see Materials and Methods for further details) were conducted. Recognition memory is composed of two distinct components: familiarity (knowledge) and recollection (remember). These components are supported by partially dissociable neural substrates within the central nervous system, with familiarity being primarily associated with the perirhinal cortex, whereas recollection mainly depends on the hippocampal formation (Brown and Aggleton, 2001). Accordingly, both the “knowledge” and the “remember” components of recognition memory were assessed using specific spontaneous behavioral tests, conducted in HFD and HFD/STZ mice at T1, T2, and T5, avoiding test-learning effects that could bias the behavioral outcomes.

Regarding the knowledge component, the NOR test was performed, and the discrimination indices (DI) and subsequently the frailty index (FI) were calculated (see Materials and Methods section for further details).

**Fig. 4:**
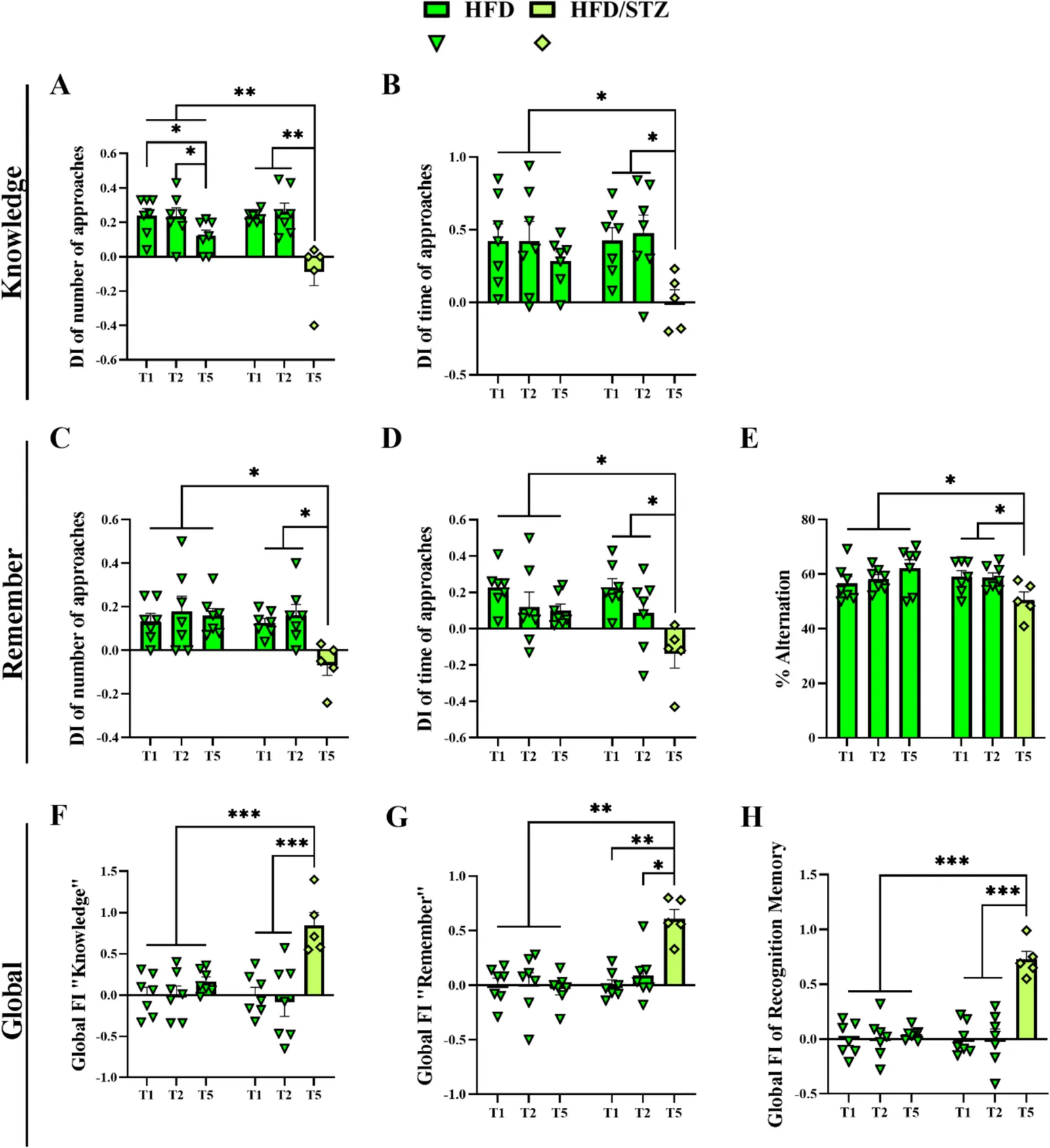
Recognition memory performances in HFD and HFD/STZ mice. Panels are organized by cognitive domain: upper row (*Knowledge*), middle row (*Remember*), and bottom row (*Global*). In the *Knowledge* row, Novel Object Recognition (NOR) parameters are shown: Discrimination Index (DI) calculated on number of approaches (**A**) and time of approaches (**B**). In the *Remember* row, Object Location (OL) parameters are shown: DI calculated on number (**C**) and time (**D**) of approaches, and Y-maze performance expressed as percentage of spontaneous alternation (**E**). In the *Global* row, Global Frailty Index (FI) scores are reported for Knowledge (**F**), Remember (**G**), and overall recognition memory (**H**). HFD mice are shown in green (triangles) and HFD/STZ mice in light green (rhombuses). Data are presented as mean ± SEM. Statistical significance (Two-Way ANOVA followed by Bonferroni *post-hoc* test): *p* < 0.05 (*); *p* < 0.01 (**); *p* < 0.001 (***)

In HFD mice, the DI for both the number (Fig. 4A) and the time of approaches (Fig. 4B) did not change at T2 (0.23 ± 0.05, and 0.42 ± 0.14, *n* = 7, respectively) compared to T1 (0.24 ± 0.04, and 0.42 ± 0.12, *n* = 7, respectively). At T5, after six months of HFD, a significant decrease in DI for the number of approaches (0.12 ± 0.04, *n* = 7) was assessed compared to T1 (*p-value* < 0.05) and T2 (*p-value* < 0.05). However, at the same time (T5) the DI of time of approaches did not significantly change in the same experimental group (0.28 ± 0.06, *n* = 7), although a decrease was evidenced without reaching a statistical significance. In HFD/STZ mice, the DI of number (Fig. 4A) and time of approaches (Fig. 4B) statistically decreased at T5 (-0.09 ± 0.08, and 0.00 ± 0.09, *n* = 5, respectively) compared to that assessed at T5 in HFD mice (*p-value* < 0.05), suggesting that an impairment of the knowledge component of recognition memory was evident after six months of HFD exposure and was significantly exacerbated by STZ treatment.

The analysis of the remember component of the recognition memory was achieved using two spontaneous behavioral tests, namely the OL and the Y-maze test.

When performed the OL test in HFD mice, the DI of the two parameters measured, i.e. the number (Fig. 4C) and the time of approaches (Fig. 4D), did not significantly change at T2 (0.18 ± 0.07, and 0.12 ± 0.08, *n* = 7, respectively) and T5 (0.16 ± 0.03, and 0.10 ± 0.03, *n* = 7, respectively) compared to T1 (0.13 ± 0.04, and 0.23 ± 0.04, *n* = 7, respectively). In HFD/STZ mice, the DI of number (Fig. 4C) and time of approaches (Fig. 4D) in the OL test statistically decreased at T5 (-0.07 ± 0.05 and − 0.14 ± 0.08, *n* = 5, respectively) compared to HFD mice at the same time (T5 HFD, *p-value* < 0.05). In the Y-maze test, the percentage of alternation triplets was measured. In HFD mice, the percentage of alternation did not change at T2 (58.24 ± 1.60%, *n* = 7) and T5 (62.18 ± 3.11%, *n* = 7) compared to T1 (56.56 ± 2.56%, *n* = 7, Fig. 4E), therefore indicating that the remember component of recognition memory remained unchanged in both spontaneous behavioral tests after six months of HFD diet.

In HFD/STZ mice, a statistically significant decrease in the percentage of alternation was observed at T5 (50.53 ± 2.96%, *n* = 5), compared to HFD mice at the same timepoint (T5 HFD, *p-value* < 0.05; Fig. 4E).

In conclusion, Y-maze results were consistent with OL test findings and indicated an early alteration of the remembering component of recognition memory in HFD/STZ mice two months after STZ induction, whereas HFD alone had no effect.

For all selected parameters and for each test performed, the FI was calculated. The global cognitive FI of recognition memory represents an integrated measure of recognition memory, combining the “knowledge” (NOR) and “remember” (OL and Y-maze) components and provides a global estimate of cognitive decline, with higher values indicating greater impairment. In particular, a global FI of the “knowledge” component was obtained using the NOR test (Fig. 4F), whereas a global FI of the “remember” component was obtained using both OL and Y-maze tests (Fig. 4G). Finally, the frailty indexes calculated for the two complementary components of recognition memory (knowledge and remember) were combined to generate a global Recognition Memory Frailty Index, reflecting the overall integrity of recognition memory (Fig. 4H).

These data revealed that HFD did not significantly affect the global FI of either the knowledge or the remember component, nor were any significant effects observed in the global FI of recognition memory (Fig. 4F, G, and H, respectively). Notably, in HFD/STZ mice, a statistically significant increase in the global FI of knowledge component was observed at T5 (0.84 ± 0.16, *n* = 5) compared to T5 in HFD mice (0.17 ± 0.06, *n* = 7; *p-value* < 0.001; Fig. 4H). For the remember component, in HFD/STZ mice, a statistically significant rise in the global FI was observed at T5 (0.61 ± 0.09, *n* = 5) compared to HFD mice evaluated at the same timepoint (T5 HFD: -0.04 ± 0.05, *n* = 7, *p-value* < 0.01, Fig. 4G). Accordingly, in HFD/STZ mice, a statistical increase in the global FI of recognition memory was gauged at T5 (0.72 ± 0.07, *n* = 5) compared to HFD mice at the same experimental time (T5 HFD: 0.06 ± 0.03, *n* = 7, *p-value* < 0.01, Fig. 4H).

### Histological differences of Hippocampus in HFD and HFD/STZ mice

Given the results obtained studying the recognition memory, the effects of the STZ induction were investigated on the hippocampus, being a critical region for spatial and recognition memory, focusing on the CA1, CA2, and CA3 subregions of the Ammon’s horn.

**Fig. 5:**
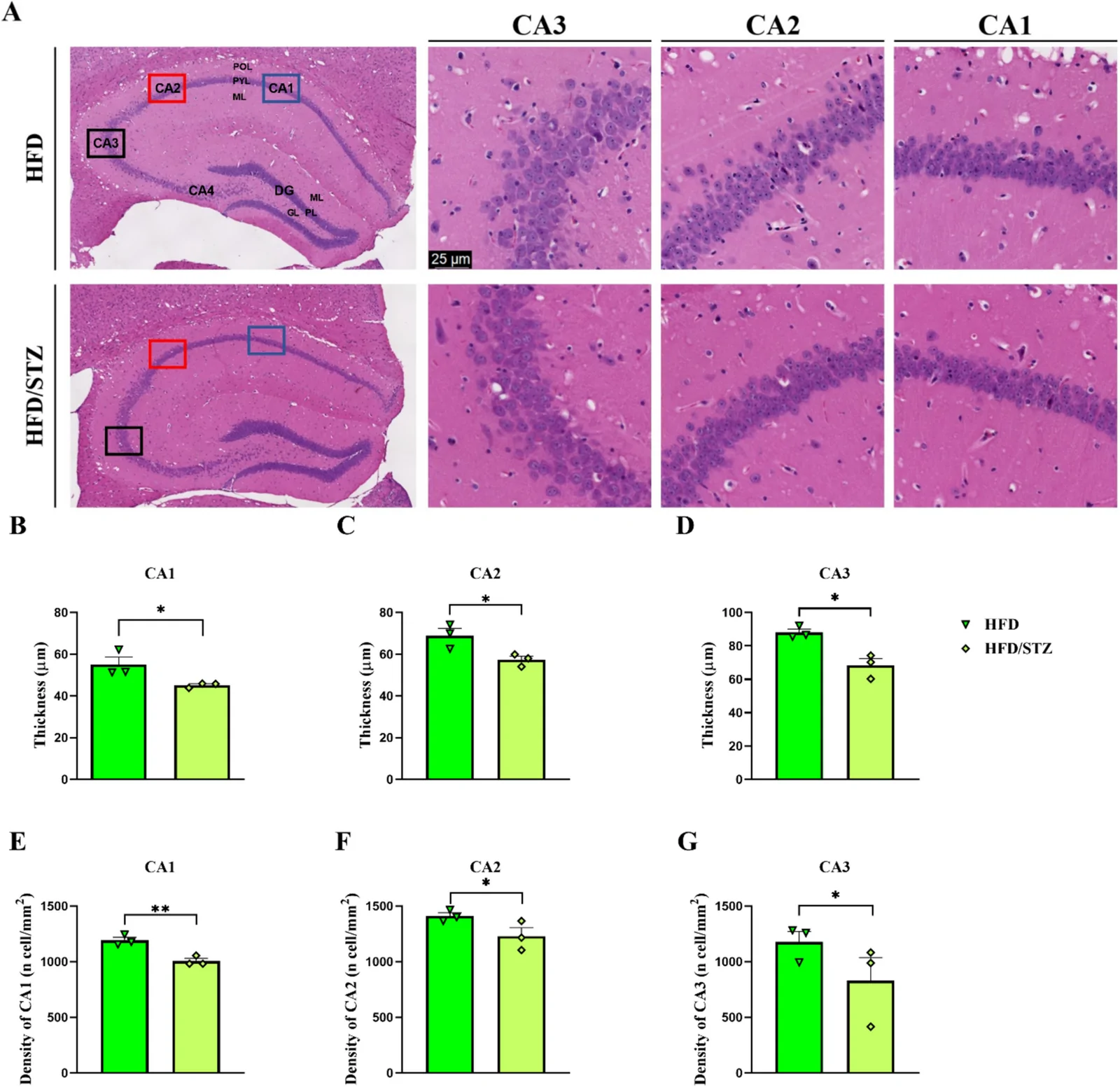
Histological characterization of the hippocampus by H&E staining. Representative brain sections showing the hippocampal cytoarchitecture (**A**): *Left column*: the whole hippocampus images, obtained with LASX Navigator, were formed of *Cornus Ammonis* show the Cornu Ammonis (CA; subdivided into CA1, CA2, CA3, and CA4), characterized by its typical three-layered organization—the polymorphic layer (POL), pyramidal cell layer (PYL), and molecular layer (ML)—as well as the dentate gyrus (DG), displaying its three distinct layers: molecular (ML), granular (GL), and polymorphic (PL) layers. Black, red, and blue squares indicate the region of interest (ROI) for CA3, CA2, and CA1, respectively. *Center and right column*: high-magnification micrographs of CA3, CA2, and CA1, respectively, from HFD and HFD/STZ mice. Light microscopy magnification: 40x. Scale bars 25 μm. Histograms showing the thickness of CA3 (**B**), CA2 (**C**), and CA1 (**D**) and the cell density measured in CA3 (**E**), CA2 (**F**), and CA1 (**G**) in HFD (green) and HFD/STZ (light green) mice. Analysis was performed on *n* = 3 mice/group (4 brain sections/animal; mean values calculated from 4–6 sampling points per subregion; for more details see Material and Methods section). Values are presented as mean ± Standard Error of the Mean (SEM). Statistical significance (One-Way ANOVA followed by Bonferroni *post-hoc* test): *p* < 0.05 (*); *p* < 0.01 (**); *p* < 0.001 (***)

Hippocampus sections were stained with H&E and analyzed by comparing brain sections from HFD and HFD/STZ mice. Figure 5A showed representative micrographs of samples. Although the overall architecture of the hippocampus appeared preserved in both experimental groups, specific changes were noted in the CA1, CA2, and CA3 subregions.

Anatomically, the CA region is subdivided into four fields, CA1, CA2, CA3, and CA4, while the dentate gyrus (DG) displays its typical V-shaped organization encompassing the CA4 area. The CA fields showed the tri-laminar arrangement, consisting of the polymorphic layer (POL), the pyramidal cell layer (PYL), and the molecular layer (ML). Similarly, the dentate gyrus exhibited its characteristic three-layered architecture, including the molecular layer (ML), the granule cell layer (GL), and the polymorphic layer (PL), with the latter corresponding to the hilus.

Measurements of layer thickness revealed significant reductions of about 20% in the CA1, CA2, and CA3 regions of HFD/STZ mice compared to HFD controls (*p-value* < 0.05 for all, Figs. 5B-D). Similarly, cell density analysis indicated a notable decrease in the CA1 (*p-value* < 0.01; reduction of 23.18%), CA2 (*p-value* < 0.05; reduction of about 30%) and CA3 (*p-value* < 0.05; reduction of about 13%) regions in HFD/STZ mice compared to HFD mice (Figs. 5E-G).

### HFD/STZ determine the p-Tau and β-Amyloid accumulations in Hippocampus

**Fig. 6:**
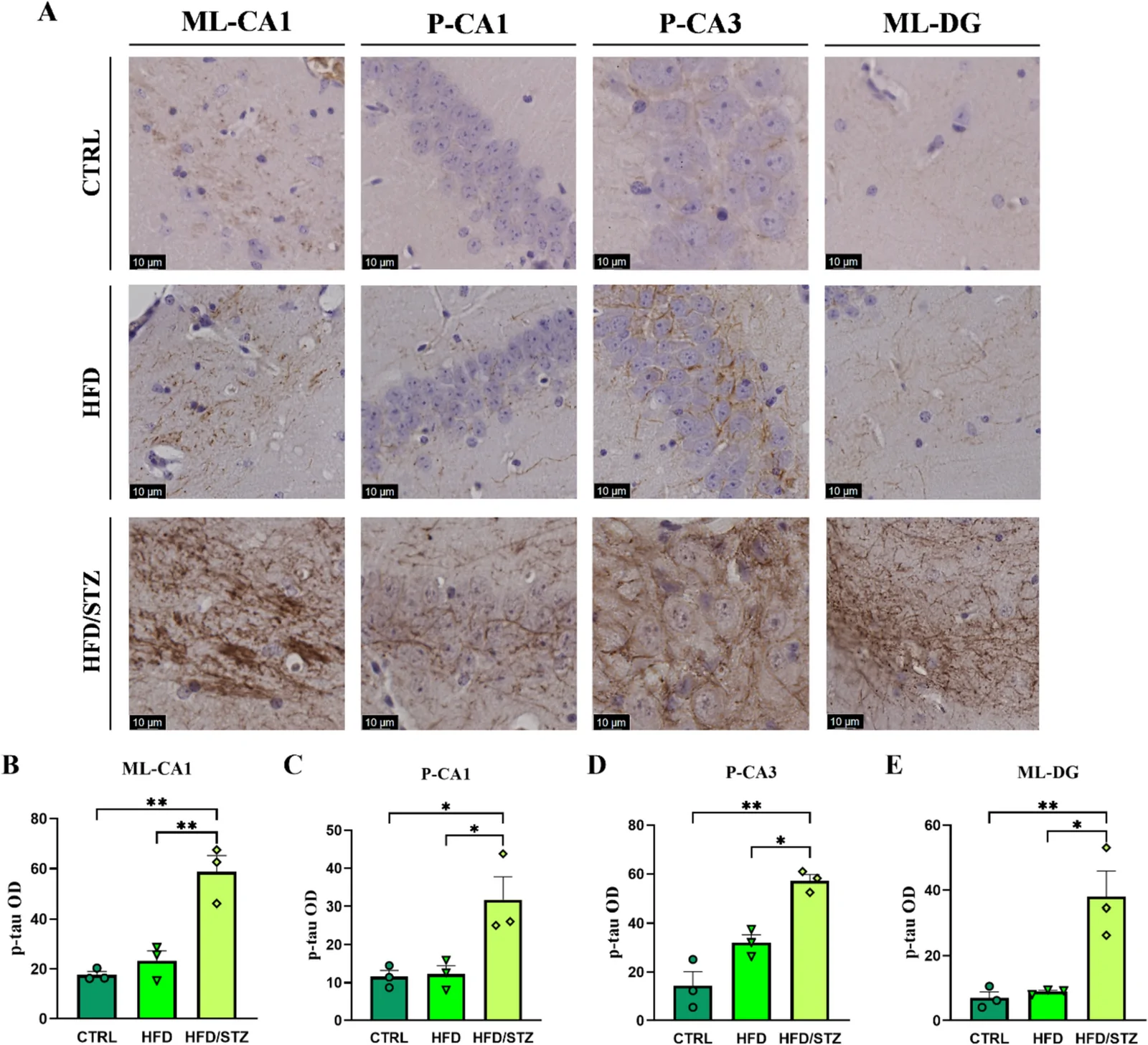
Immunohistochemical reactions for hyperphosphorylated Tau (p-Tau) (Ser235). Panel (**A**): Representative micrographs of Molecular layer of CA1 (ML-CA1), Pyramidal layer of CA1 (P-CA1), Pyramidal layer of CA3 (P-CA3), and Molecular layer of Dentate gyrus (ML-DG) from CTRL (upper row), HFD (central row), and HFD/STZ (bottom row) mice. Scale bars 10 μm. Panel (**B-E**): Histograms showing the Optical density (OD) of p-Tau signals in ML-CA1 (B), P-CA1 (C), P-CA3 (D), ML-DG (E) in CTRL (dark green), HFD (green), and HFD/STZ (light green) mice. Analysis was performed on *n* = 3 mice/group (4 brain sections/animal; mean values calculated from 10 sampling points per subregion; for more details see Material and Methods section).Values are presented as mean ± Standard Error of the Mean (SEM). Statistical significance (One-Way ANOVA followed by Bonferroni post-hoc test): *p* < 0.05 (*); *p* < 0.01 (**); *p* < 0.001 (***)

**Fig. 7:**
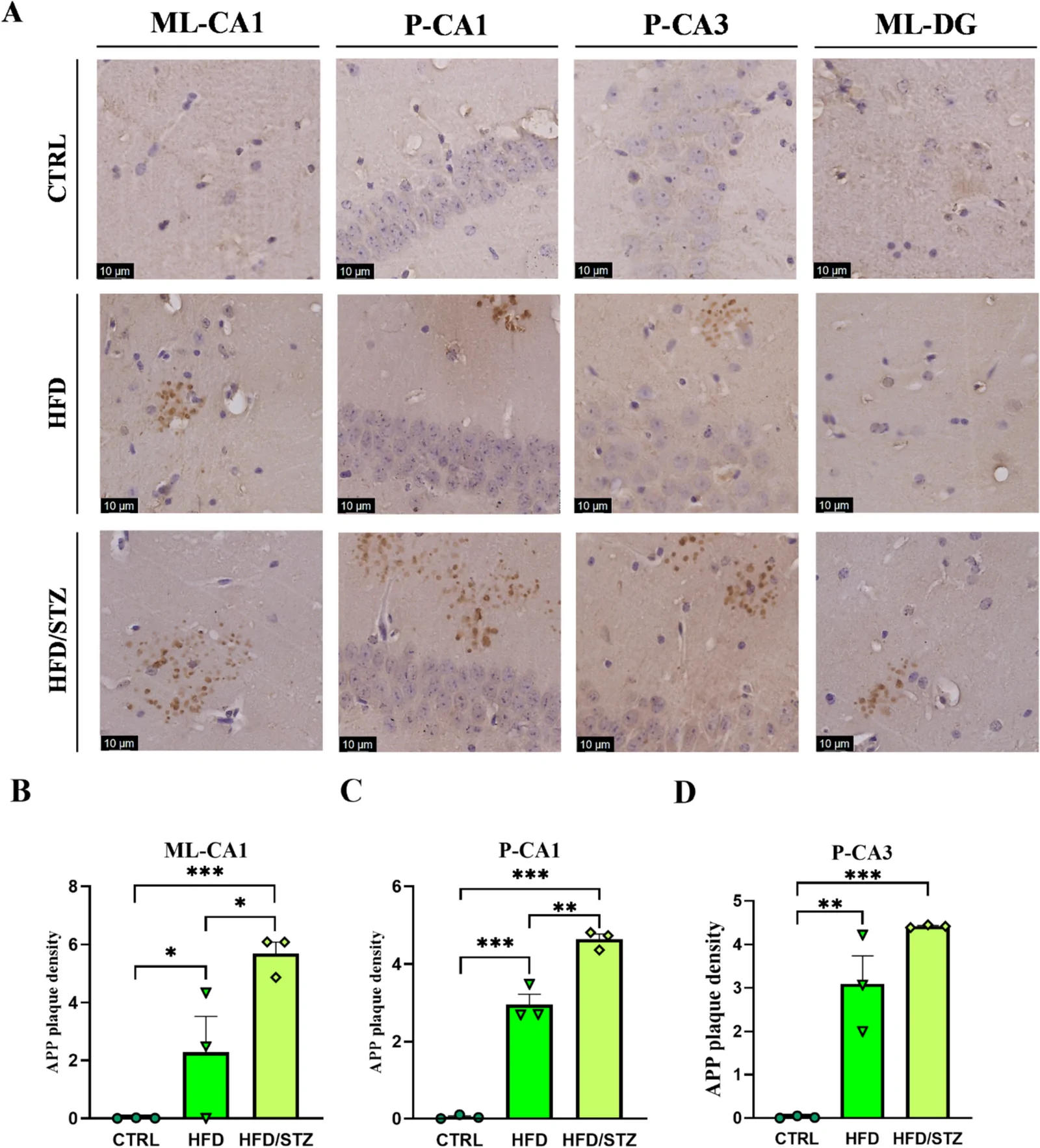
Immunohistochemical reactions for Anti-Beta (β) Amyloid. Panel (**A**): Representative images of Molecular layer of CA1 (ML-CA1), Pyramidal layer of CA1 (P-CA1), Pyramidal layer of CA3 (P-CA3), and Molecular layer of Dentate gyrus (ML-DG) from CTRL (upper row), HFD (upper row), and HFD/STZ (bottom row) mice. Scale bars 10 μm. Panel (**B-D**): Histograms showing the APP plaque density (number of APP spots/area plaque) in ML-CA1 (B), P-CA1 (C), and P-CA3 (D) in CTRL (dark green), HFD (green), and HFD/STZ (light green) mice. Analysis was performed on *n* = 3 mice/group (4 brain sections/animal; for more details see Material and Methods section). Values are presented as mean ± Standard Error of the Mean (SEM). Statistical significance (One-Way ANOVA followed by Bonferroni post-hoc test): *p* < 0.05 (*); *p* < 0.01 (**); *p* < 0.001 (***)

Immunohistochemical analyses were performed at T5 to evaluate whether HFD and HFD/STZ were associated with Alzheimer-like molecular hallmarks, specifically focusing on phosphorylated Tau (p-Tau) and β-amyloid (Aβ) in the hippocampus. p-Tau and Aβ were selected as representative markers of tauopathy and amyloid pathology, respectively, providing complementary information on neurodegeneration-related changes across experimental groups. To investigate a possible expression of these two markers in the aging brain during the senescent phase, their levels were also evaluated in a wild-type, healthy control group (CTRL, C57BL/6J male mice of the same age fed with normal diet). This approach allowed us to properly assess the specific effects of the HFD alone, as well as the changes relative to the experimental group. Representative immunohistochemical micrographs of CTRL, HFD, and HFD/STZ mice for p-tau and Aβ markers are shown in Figs. 6A and 7A, respectively.

Concerning p-Tau, no significant difference was observed between the CTRL and HFD mice (*p* > 0.05), indicating that the HFD regimen alone did not exert a significant effect on p-Tau levels. HFD/STZ mice showed a significant increase in immunopositive cell OD in the ML-CA1 region (58.73 ± 6.53; Fig. 6B) compared to CTRL (17.65 ± 1.37; *p* < 0.01), and HFD mice (23.17 ± 4.05; *p* < 0.01). Elevated p-Tau levels were also observed in P-CA1 and P-CA3 (Fig. 6C-D), with HFD/STZ mice displaying higher concentrations (P-CA1: 31.33 ± 6.50, P-CA3: 57.19 ± 2.54) compared to CTRL (P-CA1: 11.54 ± 1.69, *p* < 0.05; P-CA3: 14.27 ± 5.76, *p* < 0.01), and HFD (P-CA1: 12.21 ± 2.27, *p* < 0.05; P-CA3: 31.94 ± 3.22, *p* < 0.05). In the ML-DG region (Fig. 6E), p-Tau was significantly increased in HFD/STZ mice (37.98 ± 7.91) compared to CTRL (6.96 ± 1.90, *p* < 0.01), and HFD animals (8.89 ± 0.44, *p* < 0.05).

Regarding β-amyloid, no APP plaques were observed in CTRL mice in any hippocampal subregion (Fig. 7A). Quantitative analysis of deposit density revealed a significant and progressive increase across the experimental groups. HFD mice showed increased deposit density compared to CTRL; however, a much more pronounced and significant increase was found in HFD/STZ mice compared to both CTRL and HFD groups, localized in ML-CA1 (HFD/STZ: 4.63 ± 0.14 vs. HFD: 2.28 ± 0.93, respectively; *p* < 0.05; Fig. 7B) and in P-CA1 (HFD/STZ: 5.68 ± 0.41 vs. HFD: 3.08 ± 0.39; *p* < 0.01; Fig. 7C) regions, with HFD/STZ mice showing the highest values. Notably, any statistically significant difference was measured in the P-CA3 region (Fig. 7D) between HFD and HFD/STZ mice (4.42 ± 0.03 vs. 3.65 ± 0.57). Furthermore, Aβ deposits were not detected in the ML-DG region of HFD mice, whereas they were present in HFD/STZ mice (4.08 ± 2.11).

### Insulin resistance correlates with pancreatic damage and cognitive frailty in HFD/STZ mice

The marked hippocampal immunohistochemical changes detected in HFD/STZ mice prompted further investigation into the association between peripheral metabolic impairment and central neurodegenerative alterations.

**Fig. 8:**
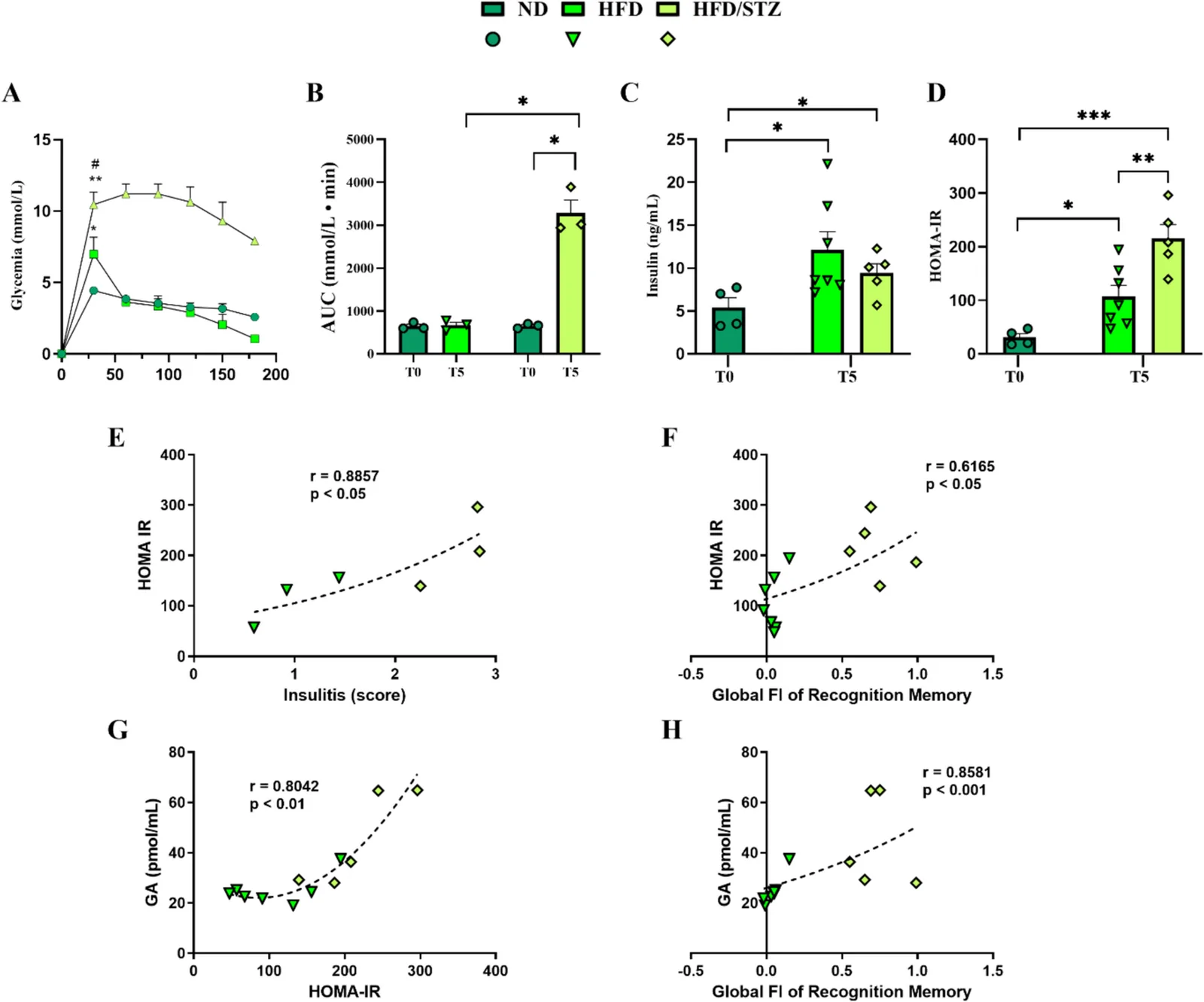
Effects of HFD and HFD + STZ on insulin resistance and correlation between metabolic parameters and cognitive decline. (**A**) Mean glucose response curves during OGTT; (**B**) area under the curve (AUC); (**C**) insulin concentrations; (**D**) HOMA-IR index at T0 in CTRL mice (dark green) and at T5 in HFD and HFD/STZ mice. Data are expressed as mean ± SEM. Statistical analysis was performed using two-way ANOVA followed by Bonferroni’s post hoc test for panels A and B, and one-way ANOVA followed by Bonferroni’s post hoc test for panels C and D: *p < 0.05; **p < 0.01; ***p < 0.001. In panel A, * indicates significance vs. ND and # indicates significance vs. HFD. Spearman’s correlations (with r and related *p-value*) were reported for HOMA IR-Insulitis score (**E**), HOMA IR-Global FI of recognition memory (**F**), GA-HOMA IR (**G**), and GA-Global FI of recognition memory (**H**)

First, an Oral Glucose Tolerance test (OGTT) was performed at T0 in CTRL mice and at T5 in both HFD (*n* = 3) and HFD/STZ (*n* = 3) mice in randomized mice. The response curve and the corresponding area under the curve (AUC) were calculated for each mouse (Fig. 8A and B, respectively).

In CTRL mice, the peak glucose level (4.44 ± 0.27 mmol/L) was reached 30 min after glucose ingestion, and then gradually declined. In contrast, the HFD group exhibited a higher peak glucose level (6.99 ± 1.19 mmol/L) compared to CTRL mice (*p-value* < 0.05, Fig. 8A). The AUC in CTRL mice at T0 was about 556.74 ± 31.99 mmol/l x min, while in the HFD group at T5, the AUC was similar (582.61 ± 45.86 mmol/L x min), showing no significant differences (Fig. 8A). The HFD/STZ group exhibited a significantly higher glucose peak level (11.21 ± 0.69 mmol/L) compared to CTRL (*p-value* < 0.01) and HFD (*p-value* < 0.05) mice, which persisted for over three hours, indicating severe impairment in glucose tolerance (Fig. 8A). The AUC in the HFD/STZ group was elevated (1702.60 ± 136.29 mmol/L x min) and significantly different compared to that assessed both at T0 in CTRL (*p-value* < 0.05) and at T5 in HFD groups (p-value < 0.05, Fig. 8B). This persistent hyperglycemia could reflect both insulin resistance and pancreatic beta-cell dysfunction.

Fasting serum insulin levels were detected in the serum of mice at T0 in four C57BL6/J mice fed with normal diet (used as baseline control) and at T5 in HFD and HFD/STZ mice. Specifically, the insulin serum levels were significantly increased in both HFD (12.10 ± 2.15 ng/mL, *n* = 7) and HFD/STZ (9.39 ± 1.10 ng/mL, *n* = 5) groups compared to CTRL mice at T0 (5.38 ± 1.16 ng/mL, *n* = 4; *p-value* < 0.05 for both groups), with no statistically significant difference between HFD and HFD/STZ groups (Fig. 8C).

Additionally, the HOMA-IR index, a measure of insulin resistance, was calculated (Fig. 8D). Both HFD (106.34 ± 21.04, *n* = 7) and HFD/STZ (214.85 ± 26.49, *n* = 5) groups showed significantly higher HOMA-IR values compared with CTRL mice at T0 (30.77 ± 7.08, *n* = 4, *p-value* < 0.05 vs. HFD; *p-value* < 0.001 vs. HFD/STZ). Notably, HOMA-IR was further increased in the HFD/STZ group compared with the HFD group (*p-value* < 0.05).

Overall, these results indicate that HFD alone induces insulin resistance, which is further exacerbated by STZ administration, leading to severe glucose intolerance and marked metabolic impairment (Abdul-Ghani et al. 2006; Tomlinson et al. 2008).

Next, to evaluate the functional relevance of pancreatic histological alterations, insulitis (expressed as weighted score, for detail see Sect.  2.7.1.1) was correlated with HOMA IR (Fig. 8E). To assess the relationship between pancreatic damage and systemic metabolic impairment, a correlation analysis was performed between the insulitis score and insulin resistance, as estimated by HOMA-IR. As shown in Fig. 8E, a strong positive correlation was observed between insulitis severity and HOMA-IR (Spearman *r* = 0.8857, *p* < 0.05), indicating that increased infiltration of pancreatic islets is associated with greater insulin resistance. These findings demonstrate that histological evidence of β-cell damage is closely linked to metabolic dysfunction, supporting the functional impact of STZ treatment on pancreatic endocrine function.

Furthermore, to better characterize the relationship between insulin resistance, metabolic parameters, and cognitive decline, Spearman’s correlation analyses were performed among HOMA-IR, glycated albumin (GA), and the global FI of recognition memory. Figure 8F, G, and H shows the positive correlation between HOMA-IR and FI scores (Spearman *r* = 0.6165, *p-value* < 0.05), HOMA- IR and GA levels (Spearman *r* = 0.8042, *p-value* < 0.01), and GA levels and FI scores (Spearman *r* = 0.8581, *p-value* < 0.001).

Overall, positive associations were observed among pancreatic damage, insulin resistance, glycation-related parameters, and cognitive impairment.

Taken together, these findings support a relationship among peripheral metabolic dysfunction, pancreatic injury, and cognitive decline, reinforcing the hypothesis that insulin resistance and glycation-related stress contribute to the progression of diabetes-associated neurodegenerative alterations.

## Discussion

Following a previously established protocol (Furman 2021; Singh et al. 2024), the present longitudinal study investigated the effects of high-fat diet (HFD)-induced metabolic dysfunction and subsequent multiple low-dose streptozotocin (STZ) administration on cognitive frailty and neurodegenerative-related alterations in a murine model of type 2 diabetes mellitus (T2DM). To better understand the metabolic and cognitive effects of the HFD and STZ induction combination in mice, the impact of HFD alone was firstly assessed. The results obtained in the present study demonstrated that the chronic exposure to HFD induced progressive metabolic dysregulation characterized by increased fasting glucose levels, body weight gain, and altered glycation-related markers, suggesting a predominant contribution of dietary exposure, rather than aging alone, to the observed metabolic phenotype (Venuti et al. 2025).

GA and MGO are two promising indicators of the glucose metabolism impairment and diabetes progression (Freitas et al. 2017; Ramachandra Bhat et al. 2019; Oliveira et al. 2024; Venuti et al. 2025). After four months of the HFD diet, fasting GA levels increased, consistent with chronic hyperglycemia. However, after six months, GA levels declined despite lasting HFD exposure and persistently elevated fasting glucose levels, suggesting a possible metabolic adaptation to prolonged dietary stress. In contrast, MGO levels decreased over time, supporting the hypothesis that serum MGO is inversely related to intracellular MGO, which can accumulate and form AGEs when the glyoxalase system is impaired (Venuti et al. 2025).

Although HFD mice developed clear metabolic alterations, STZ administration further worsened glucose intolerance and systemic metabolic imbalance, leading to a robust diabetic phenotype, characterized by a decrease in body weight, polydipsia and persistent hyperglycemia exceeding 250 mg/dL, together with elevated GA and MGO levels. Elevated GA levels indicated persistent protein glycation associated with poor blood sugar homeostatic control (Giglio et al. 2020). Concurrently, higher MGO levels suggest increased dicarbonyl stress and the subsequent formation of AGEs, which are closely involved in the development of insulin resistance, beta-cell dysfunction, and diabetic complications (Schalkwijk and Stehouwer 2020).

Histological analysis of pancreas further supports these findings, revealing significant damage in HFD/STZ mice compared to HFD mice. In particular, the HFD alone induces mild pancreatic alterations, while the STZ induction in HFD mice results in more pronounced injuries, e.g. islet damage and inflammatory infiltration. The increased frequency of high insulitis scores (grades 3–4) in the HFD/STZ group reflects severe inflammatory insult and destruction of β-cell architecture. Notably, this pancreatic inflammatory state may be associated with the establishment of a systemic low-grade inflammatory milieu characterized by the release of pro-inflammatory cytokines, that can cross the blood-brain barrier (BBB) and are known to promote neuroinflammatory responses within the hippocampus (Barrientos et al. 2015; Yang et al. 2022a; 2022b; Berends et al. 2023; Schalkwijk and Stehouwer 2020). Within this microenvironment, chronic low-grade inflammation can act synergistically with MGO and GA to activate microglial cells and astrocytes (Ibrahim et al. 2011; Leng and Edison 2020; Berends et al. 2023). Collectively, these mechanisms may be involved in the development of cognitive frailty and neurodegenerative alterations observed in the present experimental model. Accordingly, the findings of the present study support an association between the observed peripheral metabolic dysfunction and decline in recognition memory. Behavioral analyses demonstrated that severe prolonged metabolic dysfunction was associated with progressive impairment in recognition memory. HFD mice did not exhibit significant cognitive alterations, whereas HFD/STZ mice showed progressive recognition memory impairment, highlighting deficits both in the “knowledge” and “remember” components. The use of a global frailty index integrating cognitive performance across behavioral paradigms provided a comprehensive assessment of cognitive decline during aging and metabolic disease progression. Overall, these findings support the hypothesis that chronic metabolic impairment is associated with cognitive frailty and neurodegenerative-like processes. Indeed, histological and immunohistochemical analyses reinforced the relationship between peripheral metabolic dysfunction and central nervous system pathology. Given the hippocampus’s central role in learning, memory consolidation, and cognitive functions, it represents a particularly vulnerable target for metabolic insults. Its structural integrity is essential for cognitive functioning. Hence hippocampal alterations, such as those currently observed in our model, may contribute to the cognitive deficits associated with diabetes-induced brain dysfunction (Peng et al. 2021; Vilela et al. 2023).

In the models used in the present study, although the overall morphology of the hippocampus appeared normal, a detailed analysis confirmed that in the HFD/STZ group many neurons were lost, and the cytoarchitecture was damaged in areas related to recognition memory. Specifically, at cytoarchitectural level, a detailed analysis of the hippocampal structure showed significant reductions both in the thickness and cell density of the CA1, CA2, and CA3 subregions in the HFD/STZ group compared to HFD ones. Moreover, these morphological alterations were accompanied by sustained tau phosphorylation and amyloid accumulation, molecular hallmarks of Alzheimer-like pathology.

Interestingly, HFD exposure increased APP levels without significantly affecting pTau expression compared to control mice, whereas STZ administration further exacerbated APP accumulation and increased pTau expression. These findings are consistent with the hypothesis that HFD may preferentially promote amyloidogenic pathways during the early stages of neurodegeneration, whereas tau-related alterations may require prolonged exposure or additional pathological insults to become detectable. In line with this interpretation, previous evidence indicates that β-amyloid accumulation may precede overt tau pathology during neurodegenerative progression (Musiek and Holtzman 2012). Notably, although the antibody employed recognizes full-length APP rather than specific Aβ isoforms, the increased signal is consistent with altered APP processing, a hallmark of cognitive decline in diabetic and neurodegenerative conditions. While the evaluation of Aβ1–40 and Aβ1–42 would have provided additional information regarding plaque composition, the significant increase in total APP levels nevertheless supports a shift toward amyloidogenic processing in the present experimental model.

While the relatively small number of mice used for histological and immunohistochemical evaluations is a potential limitation, this was mitigated by performing multiple sections and comprehensive measurements per animal, ensuring robust data collection. Despite the small sample number of mice per group, the observed structural and molecular alterations, such as neuronal loss, APP accumulation, and tau hyperphosphorylation, demonstrated high intra-group consistency and reached statistical significance, indicating robust biological effects. Furthermore, these histological findings were strongly aligned with the behavioral and systemic metabolic deficits recorded across the entire experimental cohort.

The significant cognitive and hippocampal changes detected prompted further investigation; hence, insulin resistance was assessed and correlation analyses were performed to explore the association between peripheral metabolic impairment and central neurodegenerative alterations.

At T0, HOMA-IR values were approximately 30, consistent with insulin-sensitive mice reported in the literature (Heyward et al. 2012; Wan et al. 2022). Following HFD exposure, fasting insulin levels and HOMA-IR indices increased to values comparable to those described in established models of diet-induced insulin resistance. For instance, Heyward et al. (2012) reported fasting insulin levels of ~ 10 ng/mL and a HOMA-IR of ~ 80 in HFD-fed C57BL/6 mice, versus ~ 4 ng/mL and a HOMA-IR of ~ 30 in controls (Heyward et al. 2012). In contrast, younger mice (4 weeks old) fed an HFD for 17 weeks showed more moderate increases (8 ng/mL vs. 6 ng/mL in controls) (Meng et al. 2010). These findings suggest that both the starting age as well as the duration of dietary intervention influence the magnitude of insulin resistance and support the notion that HFD alone in the model used in this study contributes to the development of early insulin resistance.

Notably, fasting insulin levels and HOMA-IR values were significantly increased in both HFD and HFD/STZ mice, with the highest levels detected following STZ administration. These findings indicate that HFD alone is sufficient to induce insulin resistance, while STZ further exacerbates metabolic impairment and pancreatic dysfunction. Consistently, the positive correlation between insulitis severity and HOMA-IR supports an association between pancreatic inflammatory damage and systemic insulin resistance. Furthermore, the positive association between HOMA-IR and cognitive frailty suggests that insulin resistance may represent a potential link between peripheral metabolic dysfunction and central neurodegenerative alterations. The other correlation analyses performed in the present study further support the existence of a peripheral-to-central pathological *continuum*. In particular, the positive association observed between GA levels and cognitive frailty suggests that worsening glycation-related stress parallels the progression of cognitive impairment. Similarly, the correlation between HOMA-IR and GA supports the relationship between chronic metabolic imbalance and insulin resistance. Collectively, these findings support a close association between peripheral metabolic dysfunction and cognitive decline, suggesting that these alterations may represent interconnected features of disease progression.

Several mechanisms may contribute to this association. Chronic insulin resistance and hyperglycemia are known to promote oxidative stress, neuroinflammation, mitochondrial dysfunction, and altered neuronal insulin signaling, ultimately impairing synaptic plasticity and cognitive function. In addition, chronic glycation and accumulation of AGEs may exacerbate neuronal vulnerability and inflammatory responses within the hippocampus (Tomlinson and Gardiner 2008; Biessels and Reagan 2015; Maciejczyk et al. 2019; Spinelli et al. 2019). Therefore, the present findings support a relationship between diabetes-associated metabolic dysfunction and neurodegenerative alterations, potentially involving multiple interacting pathways.

Taken together, these findings provide evidence of a close association between peripheral metabolic dysfunction, pancreatic injury, hippocampal alterations, and cognitive decline in this model of type 2 diabetes. The integration of behavioral, metabolic, and histological approach reinforces the translational relevance of the HFD/STZ model for studying the complex interplay between metabolic and cognitive deterioration. From a translational perspective, these findings suggest that early identification and management of metabolic dysfunction, particularly insulin resistance and glycation stress, may represent a valuable strategy to reduce the risk or delay the onset of diabetes-associated cognitive impairment.

## Conclusion

In conclusion, this study demonstrates that the combination of HFD and STZ effectively establishes a stable metabolic profile consistent with type 2 diabetes mellitus (T2DM). While HFD alone caused weight gain, hyperglycemia, and insulin resistance, the HFD/STZ model resulted in persistent hyperglycemia, insulin resistance, elevated glycation markers (GA and MGO), and pancreatic inflammation. Changes in the metabolic profile were paralleled by marked impairments in recognition memory, affecting both the knowledge and remember components, together with significant alterations in hippocampal cytoarchitecture and increased p-Tau and amyloid accumulation. These findings highlight the association between insulin resistance and both peripheral and central complications associated with T2DM. Among the glycation-related markers analyzed, GA emerged as the parameter most consistently associated with metabolic dysfunction and cognitive impairment. Accordingly, while MGO levels did not show significant correlations, GA strongly correlated with both metabolic and behavioral alterations. Since GA reflects medium-term glycemic imbalance and protein glycation (Al-Lahham et al. 2024), these results suggest that glycation-related stress may contribute to diabetes-associated cognitive decline and that stable systemic glycation markers may reflect the progression of metabolic and neurological dysfunction in this model. Finally, the HFD/STZ model provides a robust and translationally relevant experimental platform for investigating the pathophysiology of diabetes-related cognitive decline, thereby offering a valuable tool for preclinical testing of therapeutic strategies targeting both metabolic and neurocognitive outcomes.

Future studies should focus on the molecular pathways underlying these alterations, particularly those involving advanced glycation end-products (AGEs) and neuroinflammatory responses. These results support the clinical relevance of targeting metabolic alterations at early stages of diabetes as a potential approach to prevent or mitigate cognitive decline and neurodegenerative progression. In addition, future studies should investigate the specific role of insulin resistance in the development and progression of cognitive dysfunction and determine whether such impairments share mechanistic features with early-stage Alzheimer’s disease or related neurodegenerative disorders.

## Data Availability

No datasets were generated or analysed during the current study.
